# Supplementary material for: Bacillus paralicheniformis RP01 Enhances the Expression of Growth-Related Genes in Cotton and Promotes Plant Growth by Altering Microbiota inside and outside the Root
Source: Int J Mol Sci. 2023 Apr 13;24(8):7227. doi: 10.3390/ijms24087227 (PMC10138817; doi:10.3390/ijms24087227)
Supplement: Supplementary file 1 [file ijms-24-07227-s001.zip › RP01-Supplementary Materials.pdf]

Supplementary Material

1. Supplementary Figures

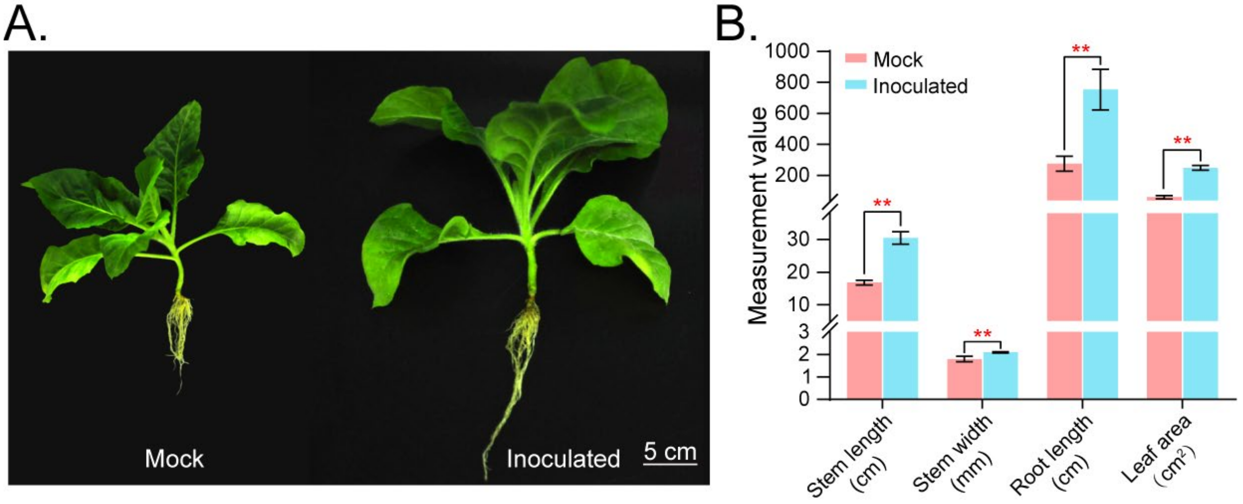

Supplementary Fig. S1 Plant phenotype inoculated with *Bacillus paralicheniformis* RP01. (**A.**) Phenotypic differences in tobacco (*Nicotiana tobacum* K326) after inoculated with RP01 and without (Mock group). Scale bar represent 5 cm. (**B.**) The biomass of pakchoi cabbage (*Brassica chinensis*) after inoculated with RP01 and without. Leaf area is measured as the area of the third leaf from the top bud. The asterisk (*) indicates a significant difference P < 0.05 and (**) indicates P < 0.01. One-way analysis of variance (ANOVA) followed by Bonferroni's post-hoc test (N=30, bars represent Mean ± Standard error)

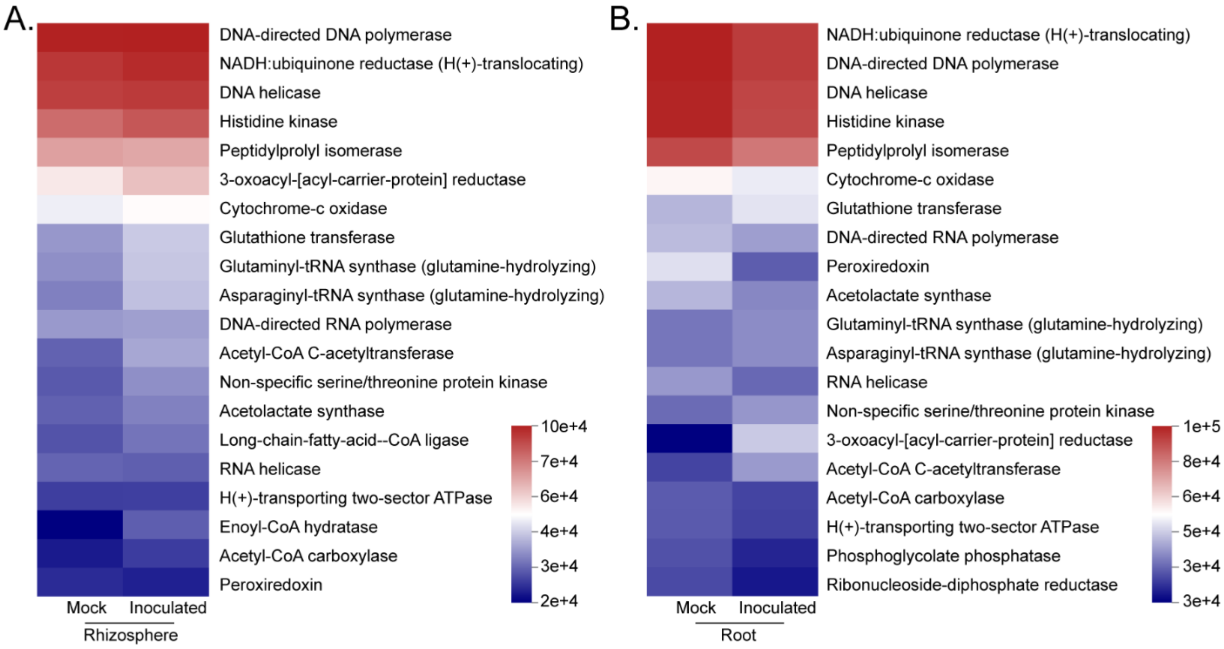

Supplementary Fig. S2 Heatmap of enzyme in KEGG by PICRUSt2 function prediction. Functional differences at rhizosphere microbiomes (**A.**) and cotton roots microbiomes (**B.**) for *B. paralicheniformis* RP01 inoculation.

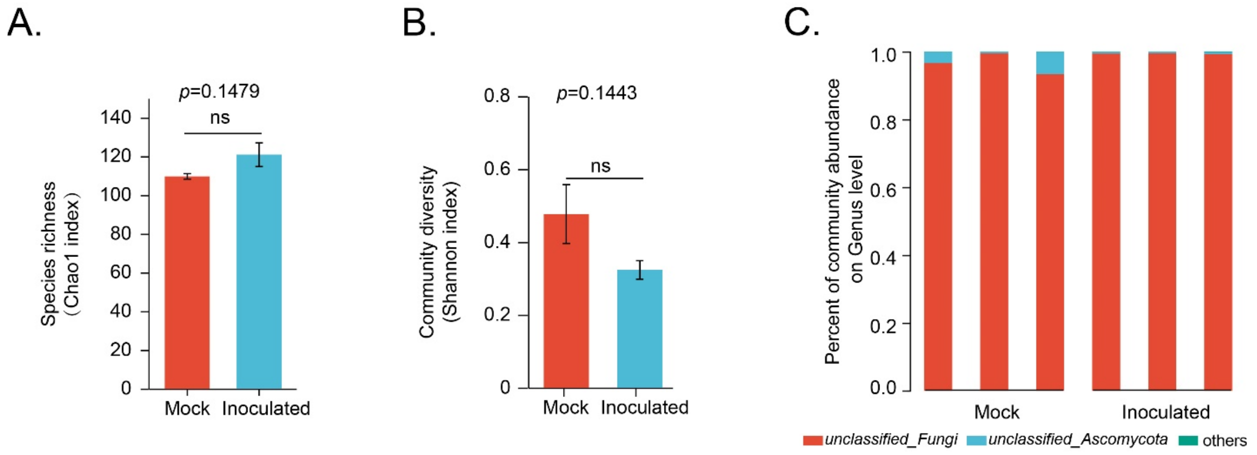

Supplementary Fig. S3 Differences in root fungal microorganism. Chao1 index for species richness (**A.**) and Shannon index for community diversity (**B.**) of the test group. Variance analysis between groups using the Student's *t*-test. "ns" means no significant difference here. Other indexes of alpha diversity were shown in **Supplementary Table S3C**. (**C.**) Relative abundance at the genus level in different samples.

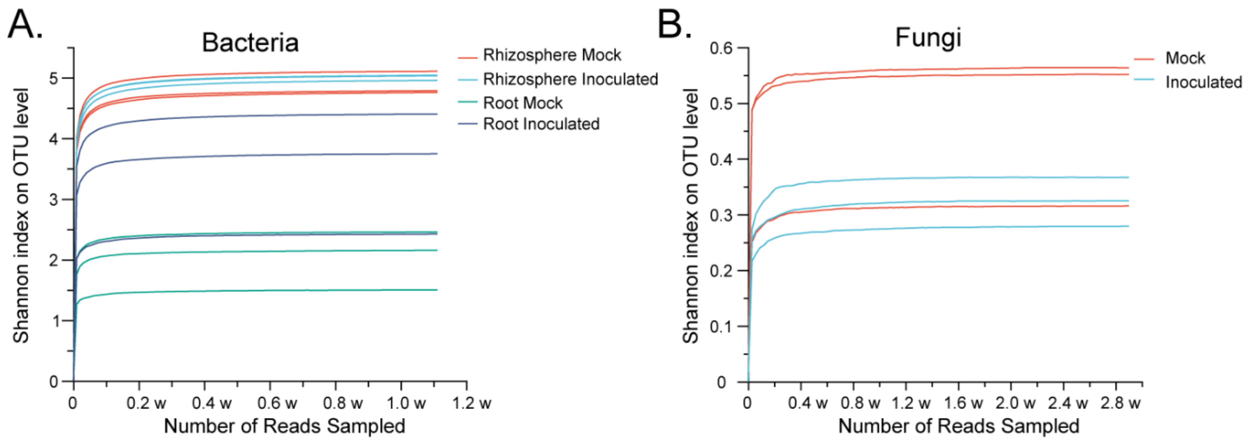

Supplementary Fig. S4 Microbiota rarefaction curves. Shannon index plot at the operational taxonomic unit (OTU) level with 97% similarity for bacteria (**A.**) and fungi (**B.**).

2 Supplementary Tables

Table S1: **The solubilization ability test of Phosphate-solubilizing bacteria.**

Strain	Soluble phosphorus content/(mg/L)	pH
CK	26.8±5.18 d	6.02 a
P1(RP01)	129±11.89 a	4.26 b
P2	45.2±5.03 c	4.38 b
P3	48.2±6.40 c	4.42 b
P4	84.9±7.65 b	4.43 b
P5	41.0±2.42 c	4.44 b
P6	45.9±5.24 c	4.30 b

Note: Different letters (a, b, and c) indicate a significant difference at $P < 0.05$, and the same letter indicate no significant difference here between groups it represents (n=3, bars represent the mean ± standard error, statistical tests: one-way ANOVA by Bonferroni's post-hoc test).

Table S2: Physiological and biochemical characteristics of *B. paralicheniformis* RP01.

Strain	RP01
Catalase test	+
Starch hydrolysis test	-
D-glucose produced acid test	+
D-xylose produced acid test	+
D-glucose produced gas test	-
methyl red test	-
Voges-Proskauer test	+
Citrate utilization test	+
gelatin liquefaction test	+
phenylalanine deaminase test	-

All test strains were cultivated for 16 h at 37 °C. Note：“+”present positive reaction，“-”present negative reaction.

Table S3: **Alpha diversity indexes after inoculted with *B. paralicheniformis* RP01.** for rhizosphere bacteria (**A.**), root bacteria (**B.**) and root fungi (**C.**). **See excel file named Supplementary file Table S3.**

Table S4: Significant differences on genus level after inoculted with *B. paralicheniformis* RP01. At rhizosphere (**A.**) and cotton roots (**B.**). **See excel file named Supplementary file Table S4**.

Table S5: **Gene annotations blasted in 6 major databases (NR, Swiss-Prot, Pfam, EggNOG, GO and KEGG).** (**A.**) Gene count statistics for six databases. (**B.**) Gene detail tables summarized from six databases. **See excel file named Supplementary file Table S5.**

Table S6: **Carbohydrate-active enzyme annotation prediction in RP01.** (**A.**) Carbohydrate-active enzyme categorical statistics in RP01. (**B.**) Gene detail tables of carbohydrate-active

enzyme prediction. **See excel file named Supplementary file Table S6.**

Table S7: **Secondary metabolites biosynthetic gene clusters prediction in RP01.** (**A.**) Statistics of secondary metabolites synthesis gene clusters. (**B.**) Gene detail table of secondary metabolites synthesis gene clusters. **See excel file named Supplementary file Table S7.**

Table S8: **Antibiotic Resistance prediction of RP01.** (**A.**) Classification statistics of antibiotic resistance genes. (**B.**) Detail table of antibiotic resistance genes. **See excel file named Supplementary file Table S8.**

Table S9: The homologous genes and unique genes in the genome of RP01 compared with the other three strains. (**A.**) The homologous genes in the genome of the four strains. (**B.**) The unique genes in the genome of RP01 compared with the other three strains. **See excel file named Supplementary file Table S9.**

Table S10: Primers for qRT-PCR.

Gene name	Forward primer (5'--3')	Reverse primer (5'-3')
Histone3	GCCAAGCGTGTCACAATTATGC	ACATCACATTGAACCTACCACTA
YUCCA4	TCTGGTCTAGCAACATCCGC	TGAGGGAAACCAAGGAGTGG
AUX1	TTAACCCTTCCATCCGCTTC	TCTCCCACACGAAGTACAATG
GA20ox	TTTACAAGAGCCGCTTGCAC	ACAAAGCCCAGCATTGTCC
GA3ox	ATGGAACCAACGCGACGATA	GTCGGGACAAGCTGGGTAAT
BES1	GGCTTGAGGCAATACGGTAATT	AGGCGGCTAGCACATCGTT
ICS1	ATGGATGAATGGGTGCGAAGG	AAGAATGCCAGAGGTAAGAGGA
EDS1	GCAGCAACAGCTCCTCTACCTCA	GGCAGACCAAGACGCTACAGATA
AOC1	CTCCCAAGCCCACAAAAGTT	ACAGAGTCCAGCCGTGATGC
OPR3	TGGAAGATGATTGTGGATGCTG	GAGATGGGTTTGTTTGTTGAGGA
TCP	CATCCGAACCCCAGATATGG	TGTAAGGGTGCTTGCAATGTG
PDF1.2	CTGTGGTAGCGGATGGTGATAAG	GTGCAGACGCATTTGCGAAGGA
ERF1	CCAAGTTCCGACAATGTTATGC	CCAACAGCTCGTCCGACAA
PAD4	GGATGGAAGAATGGAAAGAAAT	GAACTAGGAAAGCAGACTAAGG
